# Fenofibrate Administration Reduces Alcohol and Saccharin Intake in Rats: Possible Effects at Peripheral and Central Levels

**DOI:** 10.3389/fnbeh.2017.00133

**Published:** 2017-07-14

**Authors:** Mario Rivera-Meza, Daniel Muñoz, Erik Jerez, María E. Quintanilla, Catalina Salinas-Luypaert, Katia Fernandez, Eduardo Karahanian

**Affiliations:** ^1^Department of Pharmacological and Toxicological Chemistry, Faculty of Chemical and Pharmaceutical Sciences, University of Chile Santiago, Chile; ^2^Center for Biomedical Research, Faculty of Health Sciences, Universidad Autónoma de Chile Santiago, Chile; ^3^Program of Molecular and Clinical Pharmacology, Faculty of Medicine, Institute of Biomedical Sciences, University of Chile Santiago, Chile; ^4^CIB, Faculty of Health and Dentistry, Universidad Diego Portales Santiago, Chile; ^5^Research Center for the Study of Alcohol Drinking Behavior in Adolescents, Universidad Autónoma de Chile Santiago, Chile

**Keywords:** fibrates, catalase, alcoholism, treatment, peroxisome proliferator-activated receptor alpha

## Abstract

We have previously shown that the administration of fenofibrate to high-drinker UChB rats markedly reduces voluntary ethanol intake. Fenofibrate is a peroxisome proliferator-activated receptor alpha (PPARα) agonist, which induces the proliferation of peroxisomes in the liver, leading to increases in catalase levels that result in acetaldehyde accumulation at aversive levels in the blood when animals consume ethanol. In these new studies, we aimed to investigate if the effect of fenofibrate on ethanol intake is produced exclusively in the liver (increasing catalase and systemic levels of acetaldehyde) or there might be additional effects at central level. High drinker rats (UChB) were allowed to voluntary drink 10% ethanol for 2 months. Afterward, a daily dose of fenofibrate (25, 50 or 100 mg/kg/day) or vehicle (as control) was administered orally for 14 days. Voluntary ethanol intake was recorded daily. After that time, animals were deprived of ethanol access for 24 h and administered with an oral dose of ethanol (1 g/kg) for acetaldehyde determination in blood. Fenofibrate reduced ethanol voluntary intake by 60%, in chronically drinking rats, at the three doses tested. Acetaldehyde in the blood rose up to between 80 μM and 100 μM. Considering the reduction of ethanol consumption, blood acetaldehyde levels and body weight evolution, the better results were obtained at a dose of 50 mg fenofibrate/kg/day. This dose of fenofibrate also reduced the voluntary intake of 0.2% saccharin by 35% and increased catalase levels 2.5-fold in the liver but showed no effects on catalase levels in the brain. To further study if fenofibrate administration changes the motivational properties of ethanol, a conditioned-place preference experiment was carried out. Animals treated with fenofibrate (50 mg/kg/day) did not develop ethanol-conditioned place preference (CPP).In an additional experiment, chronically ethanol-drinking rats underwent two cycles of ethanol deprivation/re-access, and fenofibrate (50 mg/kg/day) was given only in deprivation periods; under this paradigm, fenofibrate was also able to generate a prolonged (30 days) decreasing of ethanol consumption, suggesting some effect beyond the acetaldehyde-generated aversion. In summary, reduction of ethanol intake by fenofibrate appears to be a consequence of a combination of catalase induction in the liver and central pharmacological effects.

## Introduction

Alcohol dehydrogenase (ADH) and mitochondrial aldehyde dehydrogenase (ALDH2) are the main enzymes involved in ethanol metabolism in the liver. However, catalase also plays an important role in the conversion of ethanol to acetaldehyde (Handler and Thurman, 1988a). Catalase is localized mainly in peroxisomes and oxygen peroxide required for its activity is provided via the peroxisomal oxidation of fatty acids (Handler and Thurman, 1988b). It was reported that oral administration of fenofibrate—a peroxisome proliferator-activated receptor alpha (PPARα) specific agonist—to rats increases catalase activity in the liver (Henninger et al., 1987; Steinberg et al., 1988; Clouet et al., 1990; Arnaiz et al., 1995; Karahanian et al., 2014). Furthermore, Karahanian et al. (2014) found that fenofibrate reduced daily ethanol intake by 70% in high-alcohol drinking UChB rats and Blednov et al. (2015) reported similar findings in fenofibrate-treated mice. It is known that the increase of systemic acetaldehyde concentration generates an aversion to ethanol consumption (Mizoi et al., 1983), and this was the basis for the treatment of alcohol-dependent individuals with disulfiram. Karahanian et al. (2014) demonstrated that the administration of fenofibrate to alcohol-drinking rats up-regulates catalase activity in the liver, leading to a higher acetaldehyde concentration in blood which ultimately produces the aversion to voluntary ethanol intake. Accordingly, they found that an oral dose of 1 g ethanol/kg produced a marked increase in blood acetaldehyde in fenofibrate-treated animals.

An alternative hypothesis also emerged to explain the effect of PPARα agonists on alcohol intake: these drugs would act in the brain where they change the expression of genes related to reward response, ultimately leading to a reduced ethanol drinking (Blednov et al., 2015). Accordingly, upregulation of several neuropeptide-coding genes in GABAergic neurons located in the amygdala (an area involved in memory consolidation and conditioning) and the prefrontal cortex (involved in the executive decision) was reported (Ferguson et al., 2014). Interestingly, upregulation of genes involved in dopaminergic transmission and downregulation of genes involved in glutamate signaling were also found (Ferguson et al., 2014), both of which important pathways related to alcohol consumption. There is strong evidence that PPARα agonists reduce the consumption of another drug of abuse, such as nicotine, decreasing its reinforcing properties in the brain (Melis et al., 2008; Mascia et al., 2011; Panlilio et al., 2012); therefore, it is plausible that a similar mechanism is also involved in decreasing ethanol intake.

In this work, we aimed at contributing to the clarification of the likely mechanisms of action of PPARα agonists on ethanol intake. Our aim was to study the peripheral (liver) and potential central effects of fenofibrate that determine its capacity to reduce ethanol consumption. Generally accepted paradigms to determine whether a drug can reduce central rewarding properties are to evaluate: (i) the prevention of conditioned place preference (CPP); and (ii) its effects on the consumption of sweet substances such as saccharin (Hajnal et al., 2004). Selectively bred alcohol-preferring UChB rats were found to consume significantly larger quantities of saccharin solution than their alcohol-avoiding counterparts (Tampier and Quintanilla, 2005). Although a common mechanism for the association between consumption of sweet solutions and ethanol intake has not been identified, this mechanism is likely involved in mediating the rewarding properties of both sweet solutions and ethanol. It has been shown that various drugs of abuse and sweet foods share the ability to increase the extracellular concentration of dopamine in the nucleus accumbens (Di Chiara, 1998; Hajnal et al., 2004) suggesting that alcohol and sweet taste may share a common dopaminergic mechanism in mediating their hedonic effects.

In this study, we administered fenofibrate to alcohol- or saccharin-drinking UChB rats, in order to establish whether the effect of this drug on the reduction of alcohol consumption is due to effects at the central level or to an increase of the aversive properties mediated by acetaldehyde generated in the liver after ethanol consumption. With this aim, we evaluated the effects of fenofibrate administration on: (i) voluntary ethanol intake; (ii) saccharin intake; and (iii) ethanol-CPP in alcohol-preferring UChB rats.

## Materials and Methods

### Animals

High-drinker UChB rats derived from the Wistar strain and bred selectively for their high alcohol intake (Mardones and Segovia-Riquelme, 1983; Quintanilla et al., 2006) were used. Two-month-old male rats (240 g ± 17 g) were housed in individual cages in temperature-controlled rooms under a regular 12-h light/12-h dark cycle. For 60 days, rats were offered a choice between 10% (v/v) ethanol solution and water, or 0.2% w/v saccharin and water. Food (Mardones rat formula, Alimentos Cisternas, Santiago, Chile) was provided *ad libitum* and the volume of water, ethanol and saccharin solutions consumed was recorded daily. After this time, 10% ethanol solution consumption stabilized at ~70 ml/kg/day, and saccharin solution mean intake was 108 ml/kg/day. All procedures used in this study were revised by and in compliance with the Bioethics Committee on Animal Research, Faculty of Medicine, Universidad de Chile (Protocol CBA0767FMUCH).

### Fenofibrate Treatment

**Figure 1:**
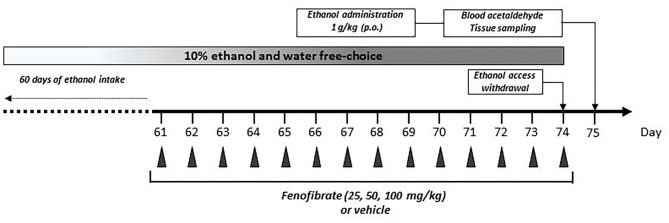
Graphical time schedule for the ethanol-drinking experiments. Twenty-eight UChB male rats were given 24 h free choice between 10% v/v ethanol and water for 60 days. Starting at day 61, 25, 50 or 100 mg/kg/day of fenofibrate or vehicle were given orally for 14 days. At the end of fenofibrate treatment, animals were deprived for ethanol access for 24 h to allow complete elimination of ethanol and acetaldehyde from the blood. At day 75, one ethanol dose (1 g/kg) was given orally, and acetaldehyde in arterial blood was measured. In the end, blood, liver and brain tissues were collected for biochemical measurements.

After 60 days of continuous free choice between 10% (v/v) ethanol solution and water (or 0.2% w/v saccharin and water), rats were divided into six groups (4 for ethanol drinking and 2 for saccharin drinking, *n* = 7 animals per group). Ethanol-drinking groups were treated with micronized fenofibrate (Fibronil^®^, Royal Pharma, Chile) administered orally as an aqueous suspension at a doses of 25, 50 or 100 mg/kg/day, respectively, or a similar volume of vehicle (water) as control for 14 consecutive days (Figure 1). Saccharin-drinking groups were treated with fenofibrate at a dose of 50 mg/kg/day or a similar volume of vehicle as control for 14 consecutive days.

In another experiment, 13 UChB rats were exposed for 59 days to a free choice between 10% (v/v) ethanol and water, animals were deprived of ethanol access on day 60 and fenofibrate (50 mg/kg, oral, *n* = 7) or vehicle (*n* = 6) was administered in a daily basis during 14 days (61–74). On day 78, a second period of free choice between 10% (v/v) ethanol and water was re-instated for 24 days (78–101). On day 102, ethanol access was suppressed again and fenofibrate or vehicle administration was repeated for 14 days (103–116). After that, a third period of free choice between 10% (v/v) ethanol and water was re-instated on day 117, which lasted for 38 days (117–154).

### Determination of Acetaldehyde in Blood

Acetaldehyde levels in arterial blood were determined as described previously (Karahanian et al., 2014). Briefly, rats under chronic ethanol consumption that were treated with fenofibrate (25, 50, 100 mg/kg/day) or vehicle for 14 days were deprived for ethanol access for 24 h to allow complete elimination of ethanol and acetaldehyde from the blood. At day 75 ethanol (1 g/kg) was given orally (as a 20% solution in saline; Figure 1). Acetaldehyde in arterial blood was measured by head-space gas chromatography at 5, 10, 15, 30 and 60 min post-ethanol administration. After blood acetaldehyde determination, animals were sacrificed by decapitation and tissues (blood, liver and brain) were sampled. Blood was collected in EDTA-tubes and centrifuged at 1500× *g* at 4°C for 10 min to obtain plasma. Liver and brain were quickly excised, weighed and stored at −80°C for posterior analysis.

### Quantification of Catalase Levels on Liver and Brain by Western Blot

Liver and brain tissues were homogenized in a pestle with 1% Triton X-100 in phosphate buffer [50 mM (pH 7.4)] containing a complete EDTA-free protease inhibitor cocktail (Pierce, Rockford, IL, USA). Cell debris was removed by centrifugation and protein content was determined with the Micro BCA protein assay kit (Pierce, Rockford, IL, USA). Samples were analyzed by western blot with an anti-catalase primary antibody (Pierce PA523246) and beta-actin (Pierce PA1183) plus a goat anti-rabbit secondary antibody conjugated to HRP (Pierce 31460). Total protein loaded per lane was adjusted to 100 μg. Blotting membranes were revealed for chemiluminiscence with Pierce ECL Western Blotting Substrate. As catalase is far more abundant in liver than in the brain, the exposure of the autoradiography films was adjusted separately to obtain clear signals in both samples.

### Conditioned Place Preference

Adult male UChB rats had free access to two bottles containing 10% ethanol and water for 60 days at the same conditions described above. Ethanol access was then restricted for 14 days, and animals were divided into two groups: one group was treated with fenofibrate 50 mg/kg p.o. for 14 days, and the other group was given the vehicle as the control (*n* = 7 animals per group). At the start of fenofibrate treatment, alcohol-induced CPP was assessed according to the methodology described by Quintanilla and Tampier (2011). Briefly, animals were placed in boxes with three compartments separated by removable guillotine doors. One compartment is painted black, the other is white and the central compartment is painted gray. The procedure had three phases: preconditioning, conditioning and post-conditioning. In the preconditioning phase, three sessions were conducted 24 h apart to determine the initially non-preferred side of the apparatus for each animal. In each session, rats were placed in the passageway leading to the conditioning chambers for 15 min. The time each rat spent in each chamber was recorded. During the conditioning phase (days 4–14), guillotine doors separated the compartments so the rats were confined to one side of the conditioning apparatus. Rats were administered ethanol orally (1 g/kg), placed immediately in their less preferred compartment and left there for 15 min. On alternate days, the animals were administered saline and placed in their preferred compartment. As a result of this conditioning schedule, ethanol has been paired five times with the less preferred compartment. The post-conditioning phase began 24 h after the last conditioning trial: a 15 min choice test was performed with no administrations in which the rats could move freely between the two chambers, and the time spent by each rat in the drug-paired compartment was recorded. Data are expressed as percentage of total time spent in the ethanol-paired compartment.

### Determination of Serum Transaminases

After treatment with fenofibrate or vehicle for 14 days, blood was extracted to determine the levels of alanine transferase (ALAT) and aspartate transaminase (ASAT) to assess liver damage (ALAT/GPT and ASAT/GOT Kits, Valtek Diagnostics, Chile). The activity was calculated as International Units/liter (IU/L), according to the indications of the supplier.

### Statistical Analyses

Data are expressed as means ± SEM. Statistical differences are analyzed by Student’s *t*-test or two-way analysis of variance (ANOVA) followed by Bonferroni’s multiple comparison *post hoc* test. A level of *p* < 0.05 was considered for statistical significance.

## Results

### Effect of Fenofibrate Administration on Voluntary Intake of Ethanol and Blood Acetaldehyde Levels

**Figure 2:**
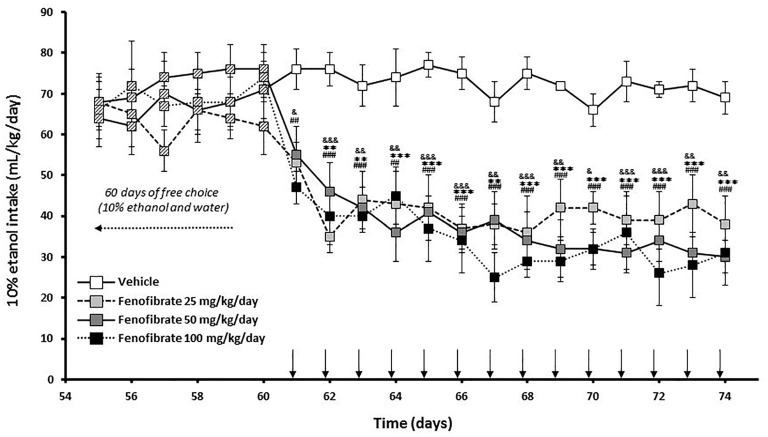
Ethanol voluntary intake of fenofibrate-treated animals. UChB rats were given 24 h free choice between 10% v/v ethanol and water for 60 days. Starting at day 61, 25, 50 or 100 mg/kg/day of fenofibrate (*n* = 7) or vehicle (*n* = 7) were given orally for 14 days (arrow). Ethanol intake was recorded daily, every 24 h. Deviations shown are SEM. two-way ANOVA test indicates that alcohol intake in the fenofibrate-treated groups was significantly lower than in the control group (^&^*p* < 0.05, ^&&^*p* < 0.01, ^&&&^*p* < 0.001 for fenofibrate 25 mg/kg/day vs. vehicle; **p < 0.01, ***p < 0.001 for fenofibrate 50 mg/kg/day vs. vehicle; ^##^*p* < 0.01, ^###^*p* < 0.001 for fenofibrate 100 mg/kg/day vs. vehicle).

Twenty-eight male UChB rats were allowed to voluntarily drink ethanol 10% (v/v) or water for 60 days until consumption was stabilized at ~70 ml ethanol (10%)/kg/day. The mean daily ethanol consumption of the animals between 1 day and 60 days was 68.9 ± 1.4 mL of 10% ethanol/kg/day. Starting at day 61, animals were divided into four groups (*n* = 7 animals per group) given fenofibrate at a daily oral dose (25, 50 or 100 mg/kg/day) or vehicle for 14 days. The effect of repeated administration of fenofibrate on ethanol intake was evaluated using a 24 h continuous-access paradigm. As shown in Figure 2, ethanol intake quickly decreases after fenofibrate administration, attaining 60% reduction after 2 days for all doses of fenofibrate (*F*_(3,336)_ = 127.0, *p* < 0.001 for drug effect). This decrease remained constant throughout the remaining days of fenofibrate treatment. There were no statistically significant differences between fenofibrate groups (*F*_(2,252)_ = 3.755, *p* > 0.05 for drug effect).

**Figure 3:**
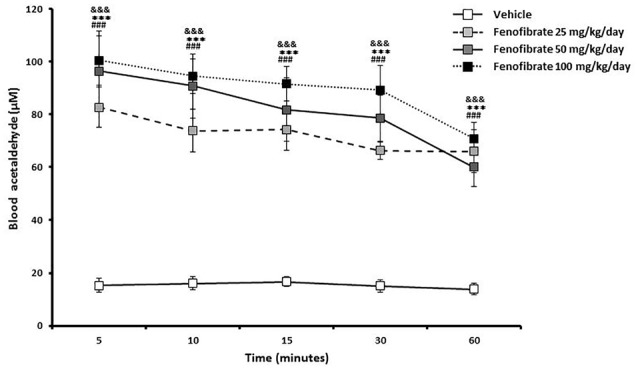
Blood acetaldehyde levels in fenofibrate-treated animals after a single oral dose of ethanol. UChB rats were allowed to voluntarily drink 10% v/v alcohol and water for 60 days on a 24-h basis. On the subsequent 14 days, 25, 50 or 100 mg/kg/day fenofibrate was given orally to three groups (*n* = 7) while the control group (*n* = 7) was given vehicle. Thereafter, animals were deprived from ethanol access for 24 h and ethanol (1 g/kg) was administered orally to both fenofibrate-treated and control (vehicle) animals. Blood samples for acetaldehyde measurement were drawn from the carotid artery at different times. Deviations shown are SEM.; two-way ANOVA test indicates that blood acetaldehyde levels in the fenofibrate-treated group were significantly higher than in the control group (^&&&^*p* < 0.001 for fenofibrate 25 mg/kg/day vs. vehicle; ***p < 0.001 for fenofibrate 50 mg/kg/day vs. vehicle; ^###^*p* < 0.001 for fenofibrate 100 mg/kg/day vs. vehicle).

Figure 3 shows that 5 min after the administration of an oral ethanol dose (1 g/kg) to fenofibrate-treated UChB rats, a marked 5-fold elevation of acetaldehyde levels in arterial blood was verified for the three fenofibrate doses tested in comparison to vehicle treated rats (from 15 μM up to 80–100 μM, *F*_(3,120)_ = 90.87, *p* < 0.001 for drug effect). A two-way ANOVA analysis of time-course acetaldehyde levels for fenofibrate groups showed that they were statistically different (*F*_(2,90)_ = 4.330, *p* < 0.05 for drug effect), but a posthoc test of individual time-points showed no significant differences between them. Blood acetaldehyde levels remained significantly elevated in fenofibrate-treated rats beyond 1 h after ethanol administration.

**Figure 4:**
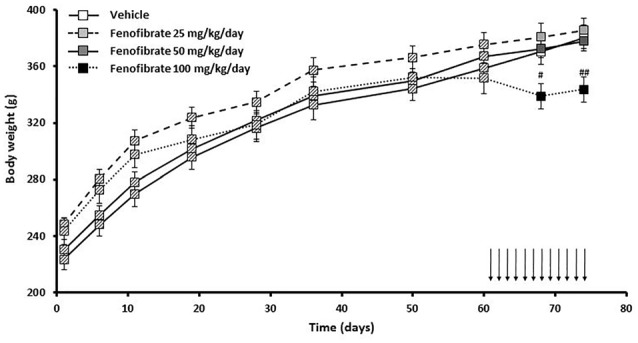
Body weight evolution of fenofibrate-treated animals. Body weight was measured weekly in the group of UChB rats used to study the effects of fenofibrate on voluntary ethanol intake. Arrows indicate the daily administration of fenofibrate (25, 50 or 100 mg/kg/day) or vehicle to the animals. Deviations shown are SEM (^#^*p* < 0.05, ^##^*p* < 0.01 for fenofibrate 100 mg/kg/day vs. vehicle).

Figure 4 shows the evolution of body weight of the same group of UChB rats used to study the effects of fenofibrate on voluntary ethanol intake. Results show that fenofibrate administration at doses of 25 and 50 mg/kg/day did not induce changes in animal body weight compared to the vehicle group (*F*_(2,36)_ = 0.7778, *p* > 0.05 for drug effect). By contrast, administration of 100 mg/kg/day of fenofibrate reduced body weight compared to vehicle treated animals (*F*_(1,24)_ = 16.44, *p* < 0.001 for drug effect). Animals showed no signs of sickness after fenofibrate treatment. Fenofibrate-treated animals compensate their decreased consumption of ethanol solution by increasing water intake (data not shown).

### Effect of Fenofibrate Administration on Voluntary Intake of Saccharin

**Figure 5:**
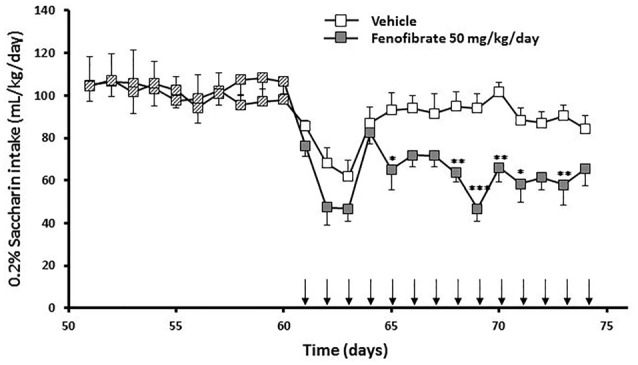
Saccharin consumption of fenofibrate-treated animals. UChB rats were allowed to voluntary drink 0.2% w/v saccharin and water for 60 days on a 24-h basis. After this period, 50 mg/kg/day of fenofibrate (*n* = 7) or vehicle (*n* = 7) was given orally for 14 days (arrows). Saccharin intake was recorded daily, every 24 h. Deviations shown are SEM.; two-way ANOVA test indicates that saccharin intake in the fenofibrate-treated group was significantly lower than in the control group from day 65 onwards (*p < 0.05, **p < 0.01, ***p < 0.001).

Taking into account that the fenofibrate dose of 25 mg/kg/day achieved the lower reduction of ethanol consumption (Figure 2) and lower blood acetaldehyde levels (Figure 3) and that fenofibrate dosing of 100 mg/kg/day produced alterations in the body weights of the animals (Figure 4), we decided to continue the following experiments with a dose of 50 mg/kg/day. In the case of saccharin consumption, animals were offered a free choice between 0.2% w/v saccharin solution and water, from two graduated bottles. After 60 days, 0.2% saccharin consumption averaged 108 ml/kg/day. The daily saccharin consumption prior to fenofibrate administration (days 1–60) was not different between the two groups (*F*_(1,720)_ = 0.48, *p* = 0.48; Figure 5). At that time, fenofibrate (50 mg/kg/day, p.o.) or vehicle were administered for 14 days, as described in Materials and Methods. As it can be seen in Figure 5, saccharin consumption was reduced in the first 3 days of fenofibrate or vehicle administration in both groups. After those first 3 days, saccharin intake returned to pre-treatment levels in the control group (the apparent difference between pre- and post-vehicle administration is not statistically significant (*F*_(1,280)_ = 2.6, *p* > 0.05). However, in the fenofibrate-treated group, saccharin consumption raised only up to 65% of pre-treatment levels (*F*_(1,140)_ = 36.25, *p* < 0.0001). Fenofibrate-treated animals compensate their decreased consumption of saccharin solution by increasing water intake (data not shown).

### Effect of Fenofibrate on Catalase Levels in Liver and Brain

**Figure 6:**
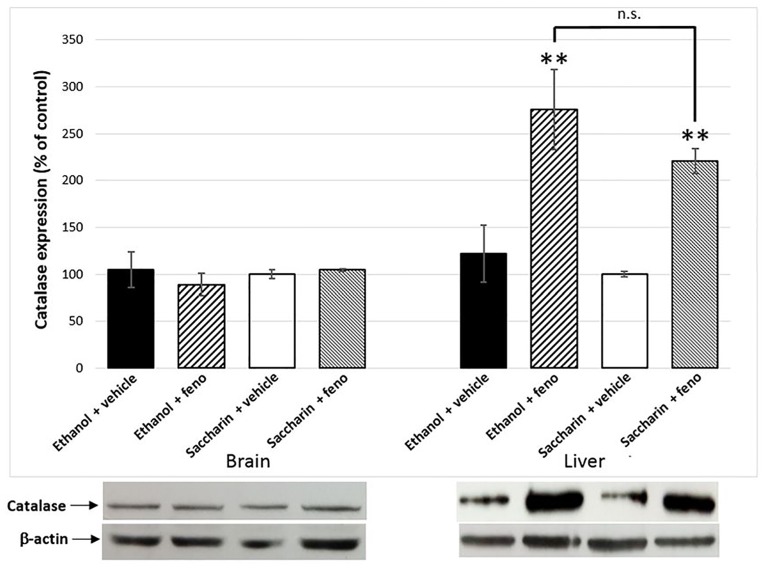
Catalase expression in liver and the brain of fenofibrate-treated animals. Catalase levels were determined by western blot in brain and liver of ethanol or saccharin drinking rats (treated with fenofibrate or vehicle; *n* = 7 for each group). Catalase levels were normalized against β-actin. Bars represent the normalized densitometric quantification of the bands. Catalase expression of the saccharin group without fenofibrate administration was used as control (100%). **p < 0.01 with respect to control; n.s. = difference statistically non-significant.

One of the effects of the administration of fenofibrate is the increase of catalase activity in the liver. In previous studies, we observed a 2.5-fold increase in liver catalase activity in fenofibrate-treated animals (Karahanian et al., 2014). As catalase-generated acetaldehyde in the brain is rewarding rather than aversive (Karahanian et al., 2011; Israel et al., 2015), it was also important to test if fenofibrate increases catalase levels in this organ. Western blot analysis showed ~2.5-fold increase of catalase in the liver of fenofibrate-treated animals (Figure 6). Conversely, catalase levels were unaltered in the brain (Figure 6). Chronic ethanol or saccharin consumption by UChB rats did not induce significant changes in catalase levels in neither the liver nor brain.

### Effect of Fenofibrate on Ethanol-Induced Conditioned Place Preference

**Figure 7:**
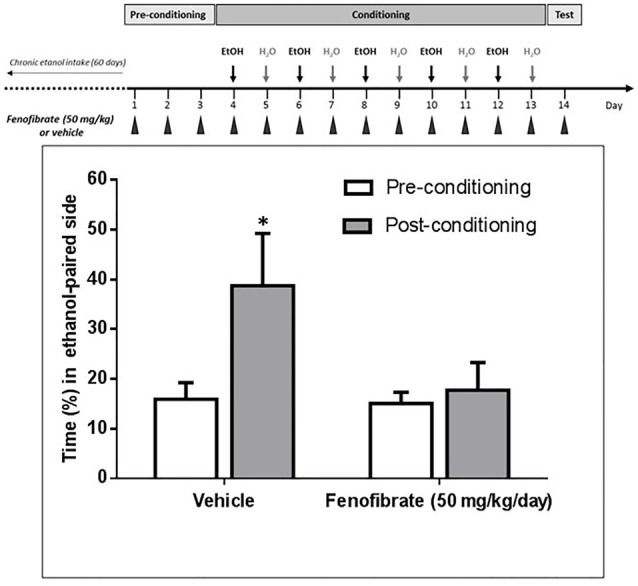
Fenofibrate administration prevents ethanol-induced conditioned place preference (CPP) in UChB rats. CPP was not generated by ethanol administration (1 g/kg oral) in UChB rats treated with fenofibrate. In the control group, CPP was clearly developed. Values represent mean time (±SEM) spent in the ethanol-paired side during the pre-conditioning and post-conditioning phases (*p < 0.05). *n* = 7 per group.

It has been reported that after 60 days of voluntary alcohol drinking, UChB rats develop ethanol-induced CPP when administered daily doses of 1 g/kg ethanol (Quintanilla and Tampier, 2011). As acetaldehyde generated by the upregulation of catalase would produce an ethanol-avoiding effect, we expected that fenofibrate would affect ethanol-induced CPP in UChB rats. As shown in Figure 7, animals treated with fenofibrate or vehicle did not present any significant difference in the time spent in the less preferred side before conditioning (15.1 ± 2.3 vs. 15.9 ± 3.9% of time; *t*-test: *t* = 0.18, *df* = 12, *p* = 0.85, white bars). On alternate days, animal received five conditioning sessions on which ethanol (1 g/kg, p.o.) was administered and placed for 15 min in the less preferred side. The postconditioning session revealed that rats treated with vehicle developed a marked CPP, expressed as a 2.4-fold increase in the time spent in the ethanol-paired compartment compared to the preconditioning value (15.9 ± 3.9 vs. 38.7 ± 12.1% of time; *t*-test: *t* = 1.8, *df* = 12, *p* < 0.05). The postconditioning time spent in the ethanol-paired compartment by fenofibrate-treated rats showed that fenofibrate prevented the development of ethanol-induced CPP (14.7 ± 2.6 vs. 18.8 ± 6.3% of time; *t*-test: *t* = 0.6, *df* = 12, *p* = 0.27).

### Effect of Fenofibrate Administration during Periods of Alcohol Withdrawal

**Figure 8:**
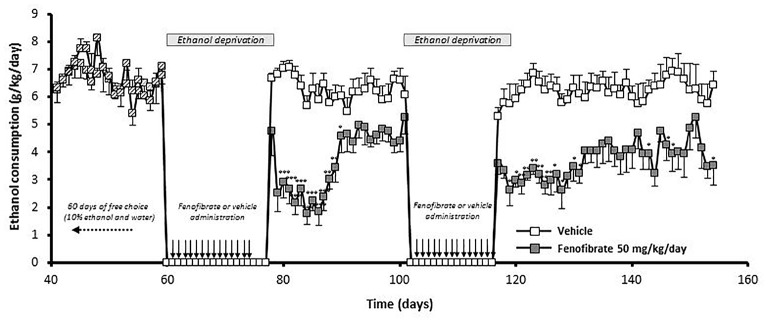
The administration of fenofibrate in periods of alcohol deprivation elicit a persistent reduction of post-deprivation ethanol consumption. Rats that consumed alcohol chronically for 60 days, were subjected to two cycles of ethanol withdrawal plus fenofibrate administration followed by re-access to ethanol without fenofibrate administration. Fenofibrate-treated animals reduced significantly its voluntary ethanol intake compared to vehicle-treated animals (two-way ANOVA test; *p* < 0.0001). Black arrows indicate the administration of fenofibrate (50 mg/kg/day) or vehicle to the animals. Deviations shown are SEM (*p < 0.05; **p < 0.01; ***p < 0.001).

We were also interested in investigating whether fenofibrate treatment would have any medium or long-term action on ethanol consumption after its administration. To do this, UChB rats were exposed for 60 days to a free-choice between 10% ethanol and water, after that animals were withdrawn from ethanol access (18 days) and at that time the administration of fenofibrate was started at a daily dose of 50 mg/kg/day for 14 days. Subsequently, the access to ethanol was restored for 24 days. Figure 8 shows that after re-access, animals treated with fenofibrate markedly reduced its mean voluntary ethanol intake by 42% compared to animals treated with vehicle (3.7 ± 0.2 vs. 6.3 ± 0.1 g ethanol/kg/day; two-way ANOVA, *F*_(1,264)_ = 288.2, *p* < 0.0001). A Bonferroni’s *post hoc* test shows that reduction of ethanol intake was statistically significant during the first 12 days of ethanol re-access.

At that time, a second cycle of deprivation/reaccess was performed, depriving alcohol and administering fenofibrate for 14 days, then withdrawing fenofibrate and allowing free consumption of ethanol for 38 days. As can be seen in Figure 8, after this second re-access, animals treated with fenofibrate reduced its mean voluntary ethanol intake by 41% compared to animals treated with vehicle (3.7 ± 0.1 vs. 6.2 ± 0.1 g ethanol/kg/day; two-way ANOVA, *F*_(1,418)_ = 311, 5, *p* < 0.0001). A Bonferroni’s *post hoc* test shows that reduction of ethanol intake in this second re-access was statistically significant during the first 15 days.

### Effect of Fenofibrate Treatment on Serum Transaminases

**Figure 9:**
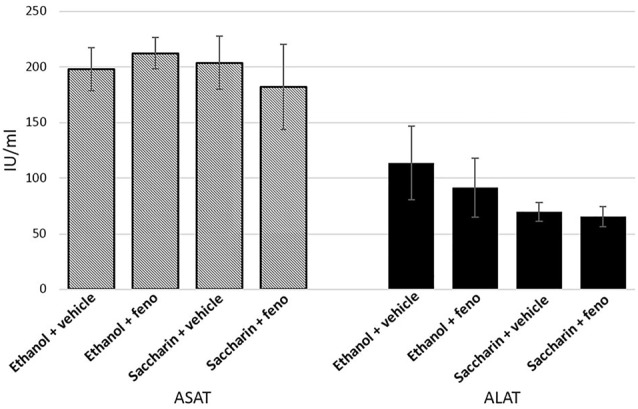
Enzyme markers of liver damage when fenofibrate was administered alone or in conjunction with ethanol. Aspartate transaminase (ASAT) and alanine transferase (ALAT) levels were determined in serum from ethanol or saccharin drinking rats (treated or non-treated with fenofibrate; *n* = 7 for each group).

The effect of fenofibrate administration in conjunction with voluntary chronic alcohol consumption on liver damage had not been studied. It was also important to determine if co-administration of ethanol and fenofibrate produce toxic effects in the liver that could affect the behavior of animals. To assess this effect, we evaluated as markers of liver damage the activity of the enzymes ASAT and ALAT. As shown in Figure 9, there are no differences in ASAT and ALAT activity between ethanol, ethanol plus fenofibrate, saccharin or saccharin plus fenofibrate treatments, indicating that no damage to the liver occurs when both ethanol and fenofibrate are administered at the same time. The apparent differences in ALAT activity between ethanol and saccharin groups are not statistically significant.

## Discussion

In a previous report, we showed that fenofibrate is able to reduce voluntary alcohol consumption by 70% in high alcohol-drinker UChB rats (Karahanian et al., 2014). We also showed that fenofibrate treatment produced a 2.5-fold increase in catalase activity in the liver and a 10-fold increase (70–95 μM) in blood acetaldehyde levels after animals were administered an oral dose of 1 g/kg ethanol. This excess of systemic acetaldehyde then would produce an aversive effect towards ethanol consumption.

However, we cannot rule out the possibility that fenofibrate exerts part of its effect at the CNS level (e.g., by altering the preference and/or the hedonistic effect of ethanol). This latter hypothesis was proposed by Ferguson et al. (2014), as they found that fenofibrate produced alterations of neuropeptide and dopaminergic gene expression in the amygdala. Indeed, fenofibrate is able to reach the brain (Weil et al., 1988; Blednov et al., 2015). With these antecedents, we had proposed that the reduction of alcohol intake seen after the administration of PPARα agonists could be a dual phenomenon: (i) PPARα activation is important in the liver, where an increase in catalase activity leads to blood acetaldehyde accumulation whose aversive effects produce a decrease in alcohol intake; and (ii) PPARα could have central effects altering neuronal circuits that are important for the ethanol drinking behavior (Karahanian et al., 2015). In order to determine the contribution of hepatic or/and CNS effects in the reduction of ethanol intake elicited by fenofibrate, we measured alcohol and saccharin intake in UChB rats after administration of fenofibrate (50 mg/kg/day). As it can be seen in Figure 2, UChB rats voluntary drink ~70 ml of 10% ethanol solution/kg/day. The next after starting the administration of a daily oral doses of 25, 50 or 100 mg/kg fenofibrate, alcohol intake decreased by 60%. This reduced alcohol consumption remained basically constant throughout the 14 days of fenofibrate treatment. This is in total agreement with data we reported in a previous work (Karahanian et al., 2014). There were no differences between the three doses of fenofibrate tested. Accordingly, the three doses of fenofibrate tested produced a similar increase in blood acetaldehyde levels after the administration of a dose of alcohol (1 g/kg) to the animals (80–100 μM vs. 15 μM in controls; Figure 3).

Interestingly, while the 25 and 50 mg/kg/day doses did not produce effects on the body weight of the animals, the dose of 100 mg/kg/day produced a decrease in this parameter (Figure 4). It is possible that this higher dose has caused discomfort, or some type of damage or simply a lower consumption of food in the animals. In any case, it has been reported that in humans only 5% of patients suffer from gastrointestinal discomfort resulting from treatment with fenofibrate (Mahley and Bersot, 2001).

In the case of saccharin-drinking rats, the administration of fenofibrate lowered saccharin consumption by 35% (Figure 5). Therefore, we suggest that a significant part of the effect of fenofibrate on the reduction in ethanol intake could take place at the central level. There is evidence that ethanol and palatable foods (e.g., sweet taste) ingestion share common mechanisms involving μ-opioid receptors and dopaminergic transmission in the brain reward system (Di Chiara, 1998; Kelley et al., 2002; Hajnal et al., 2004). Tampier and Quintanilla (2005) showed that UChB rats (bred for their high ethanol intake) have a higher preference for saccharin intake than UChA rats (low alcohol drinking). Furthermore, a long-term exposure to a 10% alcohol solution containing 0.2% saccharin induced a significant increase in alcohol consumption in UChB rats once saccharin was faded out, whereas alcohol consumption in UChA rats returned to the previous low value (Tampier and Quintanilla, 2005). In the same line, when UChB rats exposed for 3 months to a free choice between 10% ethanol and water were offered with a third bottle containing 0.2% saccharin, they maintained their levels of ethanol consumption but showing a 2.3-fold increase in their consumption of saccharin solution (Tampier and Quintanilla, 2009). These results suggest the existence of common neuronal mechanisms determining the rewarding properties of ethanol and saccharin.

While there is a direct relationship between the activation of PPARα mediated by fenofibrate with an increased catalase activity in the liver and subsequent aversion to alcohol intake due to increased levels of ethanol-derived acetaldehyde in blood, the connection between PPARα activation and the decrease in the rewarding properties of ethanol has not a straightforward explanation. PPARα is expressed throughout the central nervous system, including the midbrain (Cullingford et al., 1998) and the nucleus accumbens core and shell (Moreno et al., 2004); this last area is essential for the rewarding properties of many drugs of abuse. Ferguson et al. (2014) reported that fenofibrate produces several changes in genes related to synaptic transmission in brain regions relevant to addictive behaviors (amygdala and prefrontal cortex). However, a mechanistic relation between PPARα activation in the brain and changes in the expression patterns of those genes is still lacking. Dopamine neurons in the ventral tegmental area (VTA) express nicotinic acetylcholine receptors (Clarke et al., 1985), whose activation lead to an increased dopaminergic activity (Pidoplichko et al., 1997). It has been reported that activation of PPARα induces a yet-unidentified tyrosine kinase(s) which phosphorilates and negatively regulates β2-nicotinic acetylcholine receptors, thus decreasing the dopaminergic activity of VTA neurons (Melis et al., 2008, 2010). According to this, one possibility is that fenofibrate-mediated activation of PPARα would diminish dopamine release in the mesolimbic system, thus decreasing reward. The effect of fenofibrate at the central level would not be surprising, since it has been described that PPARα activation leads to a lower consumption of nicotine, due to a decrease of its rewarding properties (Mascia et al., 2011; Panlilio et al., 2012). In these studies, PPAR-α agonists dose-dependently decreased nicotine-induced excitation of dopamine neurons in the VTA and nicotine-induced elevations of dopamine levels in the nucleus accumbens shell of rats.

Disulfiram inhibits ALDH2 in the liver, leading to a buildup of acetaldehyde in the periphery when the individual consumes alcohol. This excess of acetaldehyde finally produces aversion to alcoholic beverages. This effect is clearly observed in UChB rats when they initiate their alcohol intake (Tampier et al., 2008); however, when these rats have ingested ethanol chronically, disulfiram although having an identical effect in elevating blood acetaldehyde levels is completely ineffective in reducing ethanol intake (Tampier et al., 2008). It is noteworthy that in these studies, acetaldehyde accumulated at even higher concentrations (150 μM) than those achieved in the studies presented here (80–100 μM). Similarly, in humans, the success rate of treatment with disulfiram is quite low because many patients do not show aversion to ethanol ingestion and further develop tolerance to disulfiram. Recent placebo-controlled clinical work and meta-analyses also show that disulfiram—as a drug (in blind studies)—is not different from placebo in reducing ethanol relapse in alcoholics (Skinner et al., 2014; Yoshimura et al., 2014). These studies might be taken as an indication that following chronic ethanol intake a systemic elevation of acetaldehyde does not inhibit ethanol consumption. However, increases in systemic acetaldehyde following the administration of an adenoviral vector (which does not enter the brain) coding for an antisense-RNA that inhibits ALDH2 synthesis or another adenoviral vector that also overexpresses ADH, markedly inhibited (50%–65%) voluntary ethanol intake of rats that had ingested ethanol chronically for 60–75 days (Ocaranza et al., 2008; Rivera-Meza et al., 2012). Rather, the lack of disulfiram effect on ethanol intake in animals fed alcohol chronically (and in alcoholics) may stem from the fact that disulfiram crosses the blood-brain barrier and also inhibits ALDH2 in the brain (Hellström and Tottmar, 1982), increasing acetaldehyde levels in this organ. A number of studies have shown that oppositely to its peripherally aversive actions, acetaldehyde possesses reinforcing and stimulating effects in the brain (Rodd et al., 2005). Thus, in ethanol-fed rats brain disulfiram might contribute an added hedonistic effect of ethanol to counter the aversive effects of acetaldehyde in the periphery. Therefore, in order to find a drug that is more effective than disulfiram in reducing alcohol consumption in patients, this drug should ideally stimulate the production of acetaldehyde in the periphery, and not in the brain. To clarify whether treatment with fenofibrate increases levels of catalase in the brain, we quantified catalase levels by western blot. Clearly, no increase in catalase is observed in the brain after the treatment with fenofibrate. In contrast, levels of catalase in the liver undergo a marked increase (Figure 6). As fenofibrate induced a reduction on voluntary ethanol intake in chronically drinker rats, we hypothesized that it can also interfere with the development of CPP. In fact, the administration of fenofibrate fully blocked CPP (Figure 7). It is interesting that fenofibrate was also able to reduce alcohol consumption when it was administered not simultaneously with ethanol (Figure 8). In animals that have been consuming alcohol for 60 days, when alcohol was withdrawn and fenofibrate was given for 14 days, a significant decrease in consumption was observed on the following days of ethanol re-access. A second cycle of deprivation/administration of fenofibrate and subsequent re-access to ethanol also showed a decrease in consumption, and this effect lasted longer than in the first cycle (12 days vs. 15 days). These observations have two possible explanations: (i) the levels of catalase in the liver may remain elevated several days after withdrawal of fenofibrate, so that the aversive reaction to peripheral acetaldehyde would continue to occur; or (ii) fenofibrate produced effects at the central level, decreasing the preference for ethanol when it was offered back to the animals. Further experiments are needed to clarify this point.

One of our concerns was that the co-administration of fenofibrate in conjunction with voluntary alcohol consumption could somehow produce liver damage. Under our experimental conditions, no changes in ASAT and ALAT were detected in the serum of ethanol-drinking fenofibrate-treated animals (Figure 9). Tsutsumi and Takase (2001) studied the effect of fenofibrate administration to rats subjected to forced consumption of alcohol (ethanol-containing liquid diet); they observed not only that fenofibrate administered together with ethanol did not produce liver damage, but that some indicators improved (ALAT decreased in serum). In addition, treatment with fenofibrate reverted the hepatic steatosis induced by the consumption of the ethanol-containing liquid diet.

In summary, fenofibrate produces a 60% decrease in voluntary ethanol intake in high-drinker rats and 35% reduction in saccharin intake. These results suggest that fenofibrate reduces ethanol appetence by a combination of peripherally (aversive) and central effects. Thus, fenofibrate administration can be further explored as a new pharmacological strategy for the treatment of alcoholism.

## Author Contributions

All authors contributed to the experiments. EK and MR-M wrote the article.

## Conflict of Interest Statement

The authors declare that the research was conducted in the absence of any commercial or financial relationships that could be construed as a potential conflict of interest.
